# *Learn!Bio*—A time-limited cross-sectional study on biosciences students’ pathway to resilience during and post the Covid-19 pandemic at a UK university from 2020–2023 and insights into future teaching approaches

**DOI:** 10.1371/journal.pone.0300824

**Published:** 2025-09-25

**Authors:** Katy Andrews, Rosalie Stoneley, Katja Eckl

**Affiliations:** Department of Biology, Edge Hill University, Ormskirk, Lancashire, United Kingdom; Khyber Medical University, PAKISTAN

## Abstract

Higher education in biosciences is substantiallyinformed by hands-on field trips and practical laboratory skills-training. With the first Covid-19 national lock-down in England in March 2020, on-campus education at higher education institutions was swiftly moved to alternative provisions, including online only options, a mix of synchronous or asynchronous blended, or hybrid adaptions. Students enrolled on an undergraduate bioscience programme have been faced with unprecedented changes and interruptions to their education. This study aimed to evaluate bioscience students’ ability to adjust to a fast-evolving learning environment and to capture students’ journey building up resilience and graduate attributes. A total of 317 Bioscience undergraduate students in years 1–3 at the biology department at a northwest English university participated in this anonymous, cross-sectional, mixed-method study with open and closed questions evaluating their perception and feedback to remote and blended learning provisions during the Covid-19 pandemic and post pandemic learning capturing academic years 2019/20–2022/23. The Covid-19 pandemic and the consequent restriction of personal social interaction resulted in an significant decrease in the mental wellbeing of undergraduate bioscience students in this study, cumulating in poor or very poor self-rating of wellbeing in spring 2021; while at the same time students showed evidence of advanced adaption to the new learning and social environment by acquisition of additional technical, social and professional graduate-level skills. Post pandemic, bioscience students worry about the increased living costs and are strongly in favour of a mixture of face-to-face and blended learning approaches. Our results show that bioscience students can self-report poor mental health while developing resilience, indicating tailored support can aid in developing students’ resilience. Students have adjusted with ease to digital teaching provisions and now expect higher education institutions continue to offer both, face-to-face, and blended teaching, reducing the burden on students’ notably risen living costs.

## Introduction

**Fig 1:**
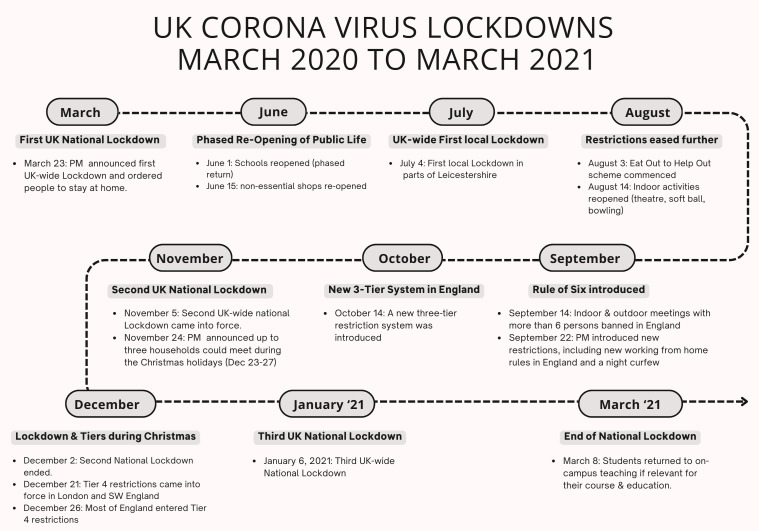
Covid-19 timeline in the UK: A comprehensive Covid-19 timeline for the United Kingdom from March 2020 to March 2021 detailing national and local restrictions and national lockdowns. In reference to this manuscript the term “pre-pandemic” captures any timepoint before the first national lockdown in March 2020, whereas post-pandemic refers to the period from January 2022 onwards (Fig 1 generated by KE based on information from reference [5]).

In March 2020 the Word-Health Organisation (WHO) declared Covid-19 a pandemic [1]. In the UK, the government legislated the first national lockdown, which commenced March 26^th^, 2020, resulting in a public standstill and a closure of all face-to-face and on-campus teaching at English universities [2,3] with most practical courses, laboratory experiments and field excursions suspended or converted to online or blended provisions for the majority of academic years 2019/2020 and 2020/2021 [4], respectively, a detailed timeline of national and local lockdowns and restrictions for the United Kingdom is shown in Fig 1

Benchmark statements for bioscience students’ education (2023) outlined by the Quality Assurance Agency for Higher Education (QAA) highlight the need for practical skills training in the laboratory, field, and computer/IT settings. During their studies, bioscience students can also expect to experience “*authentic research*” throughout their course while acquiring competencies in the lab, field, and computational practical and transferable skills essential for a post-graduate role [6]. A paper presented by Coward and Gray (2014) for the Royal Society for Biology had calculated an average bioscience student in the UK can expect 500 hours of practical training in their three years as an undergraduate student, while speciality training in the laboratory, field, or computational education might depend on the chosen specific subject [7].

The Covid-19 pandemic restricted students’ access to laboratories and research facilities, and most field trips and excursions had been cancelled. Higher education institution (HEI) providers sought alternative strategies to prepare students for a successful post-graduation career, including access to interactive online lab sessions, the introduction of lab or field virtual simulations, and lab/field activities to be conducted at home with any required material sent by post to all participating students [8–11].

With student accommodations closed and campus and social interactions significantly reduced [12,13], the Covid-19 pandemic has changed higher education teaching and learning world-wide, and has impacted students’ wellbeing and their pathway to personal and career-related resilience and skills development in a yet not fully understood extent [14,15].

This manuscript presents students’ feedback and voices from 317 participants surveyed from 2020−2023 in a cross-sectional mixed-methods study conducted at a North English university on bioscience students’ perception on their higher education during and post the Covid-19 pandemic and how the closure of laboratories and limitations to access campus facilities impacted their mental wellbeing and pathway to resilience development. Follow up surveys conducted in 2022 and 2023 post-pandemic compares students’ perception on the consequences of the pandemic on bioscience learning and career progression.

This study follows the AdvanceHE definition of mental health and wellbeing, summarised in reference [16] and the excellent definition of resilience as described by the American Psychological Association [17]. Students’ wellbeing, resilience to change and ability to grow in times of uncertainty (pathway to resilience) was evaluated against the Ten UK Measures of National Wellbeing [18].

Teaching, learning and pedagogy research at this department is informed by constructivism-informed didactic for science teaching, learning and research (Kersten Reich [19], John Dewey) and by Maria Montessori’s [20] concepts for adult learners, which is reflected in the study design.

We hypothesised (1) most bioscience students exhibit the ability to adjust to a fast-evolving learning environment and build up resilience. We also hypothesised (2) that students preferred to return to a pre-pandemic mode of teaching. Post-pandemic, students are notably supportive of a blended teaching concept but are not in favour of purely online learning.

## Methods

### Study design and study setting

This anonymous, cross-sectional, mixed-method cohort study followed a convergent parallel design with a focus on qualitative and descriptive data collection and analysis [21].

The study captured students’ feedback at four time points between November 2020 and May 2023 evaluating perception and acceptance of changes to **(1)** Learning during the pandemic and adjusting to a new challenging situation **(2)** wellbeing and resilience, and **(3)** consequences of the pandemic & students’ expectations from providers as reported by participants [22]. All participants have been in education during the Covid-19 pandemic, either at school or university level.

Our pathway to study development is shown in S1 Fig. In brief, upon closure of campus teaching and the move to remote and hybrid teaching preliminary, unstructured student feedback and comments from spring 2020 prompted an in-depth literature review and first structured student surveys throughout late spring and early summer 2020 (module level only). Extended survey questions were then established based on student feedback and internal review while taken into account questions on wellbeing and resilience from the UK Measures of National Well-being surveys [18], and the Connor Davidson Resilience Scale principles [23]. Estimated completion time for surveys S1 and S2 had been estimated with 30–45 minutes (62 and 79 questions, respectively). The student gatekeeper requested follow-up surveys are kept to a maximum completion time of 20–25 minutes. Surveys S3 and S4 entail 34 and 38 questions, respectively. A range of new questions have been added which captured the changed circumstances of lived student experiences.

All students enrolled in an undergraduate bioscience degree (years 1–3) at Edge Hill University were eligible to participate in this study. Students were invited by email which entailed a direct link to the respective JISC online survey studies. Each questionnaire was accessible on the JISC online survey platform for four weeks, with one email reminder sent two weeks after the first invitation. Participation in each study was entirely voluntary and not linked to the participation in a previous or follow-up study. We completed four studies: Study-survey S1 (November 2020, mid-pandemic I, data collected from 23/11/2020 to 08/12/2020), study-survey S2 (April 2021, mid-pandemic II, data collected from 28/04/2021 to 04/05/2021), study-survey S3 (March 2022, follow-up I, data collection from 08/03/22 at 9am to 06/04/22), and study-survey S4 (May 2023, follow-up II, data collection from 03/04/2023 to 02/05/2023). All participants in this study were 18 years of age or older and fully able to consent.

### Student cohort and participants

Cohort sizes and number of participants for each study are shown in Table 1. No personal identifiers were collected.

**Table 1:** Learn!Bio longitudinal, cross-sectional study 2020-2023: Cohort sizes and participants – overview.

Study	Date	Cohort size*	Participants	Survey length	*Completion time*
**Study 1 (S1)**	November 2020	273 students	70 participants (25% of cohort)	62 questions	35-40 minutes
**Study 2 (S2)**	April 2021	273 students	34 participants (12% of cohort)	70 questions	35-40 minutes
**Study 3 (S3)**	March 2022	311 students	130 participants (42% of cohort)	34 questions	20 minutes
**Study 4 (S4)**	May 2023	363 students	83 participants (23% of cohort)	38 questions	20-25 minutes

**Explanations:** Dates survey conducted, survey lengths and estimated time students required to complete the anonymous JISC online survey. All students enrolled in a bioscience undergraduate degree at Edge Hill University were eligible to participate. The cohort size is the total number of students at the time of the study eligible to participate.

### Quantitative and qualitative data, data interpretation

We conducted four surveys based on a convergent mixed-method approach with all surveys entailing quantitative and descriptive (single/multiple-choice questions) and extended qualitative questions (open answer questions) [24].

Upon completion of study, all raw data, each survey layout, and all questions were downloaded from the JISC online survey server and saved on a secure university cloud-based server. Qualitative and quantitative data was collected and analysed separately and combined for *joint display* of results [21]. Quantitative data from all four studies was manually analysed using Microsoft Excel while taking into consideration the grouping, as shown in Table 2. Each variable/question was analysed independently. Due to the small sample size per group and study and the overall descriptive nature of this study, data is presented in raw format,albeit in percentages, allowing easier comparison across all groups, cohorts, and surveys. Where appropriate, statistical analysis was performed with GraphPad Prism version 10 to show evidence of significance of results and conclusions

**Table 2:** Learn!Bio study: Grouping of Participants.

Study year (level)	Study 1 (Nov 20)	Study 2 (Apr 21)	Study 3 (Mar 22)	Study 4 (Apr 23)
Total number of participants	70 (100.0%)	34 (100.0%)	130 (100.0%)	83 (100.0%)
Year 1 (level 4 – L4)	26 (37.1%)	16 (47.1%)	30 (23.1%)	27 (32.5%)
Year 2 (level 5 – L5)	28 (40.0%):	11 (32.4%):	59 (45.4%)	41 (49.4%)
Year 3 (level 6 – L6)	16 (22.9%)	7 (20.6%)	41 (31.5%)	15 (18.1%)

**Explanations:** Studies 1–4 with total numbers of participants per study and grouping into years 1–3 (levels 4–6) for each study. Students’ perceptions of learning at the biology department during the Covid-19 pandemic and the following years have been analysed cross-sectional and long-term while comparing overall feedback from participants of classes of 2018–2021 (blue), 2019–2022 (yellow), 2020–2023 (green), 2021–2024 (red), and 2022–2025 (grey). Years 1–3 refer to the UK National Qualification Framework (NQF) levels L4-L6.

Quotes displayed in this manuscript are examples only and are not a full representation of all comments provided: Most free-text answer options allowed participants to expand their feedback on topics raised in previously answered quantitative questions, enabled data validation, and provided keywords for coding [25]. All free text answers were analysed individually and across all year with Excel for Microsoft 365 enterprise, version 2402: Numbers of submissions (total, per cohort) were recorded, and each submission was indexed for later identification and publication [26]. The free text responses were analysed utilising the concept of thematic analysis with open coding procedures [27]. Preliminary coding was completed on keywords which arose from the quantitative lead question followed by a detailed coding based on prompts provided by twelve emerging themes covering topics from mental and physical wellbeing, remote and on-campus teaching, staff support, coping with social anxiety and increased living costs post-pandemic (initiated and completed by KME, reviewed and corrected by RS and KA).

### Ethical approval

Ethical approval for this study was confirmed by the *Faculty of Arts and Science Research Ethics Committee* (approval reference *FREC/1920/034*) with permission to survey undergraduate students granted by the students’ gatekeeper, the head of the biology department. We asked students about their place of living, their study programme, if they had care responsibilities (and in Study S1 also about number and age of children they cared for), which could lead to indirect identification of participants [28], if combined with other personal data. For this reason, datasets on gender, sex, age, and origin have not been collected, as per FREC guidance on staff-led studies with student data.

All JISC online surveys (S1-S4) in this study entailed a detailed introduction to the study rationale outlining any ethical dilemmas and how these would be addressed, and a downloadable participant information sheet. Participants were reminded that the survey was completely anonymous, and withdrawal was not possible once the survey had been submitted. On the next online survey page, students were then asked to consent to the JISC online survey by ticking the relevant answers: *I consent to participate in this anonymous survey* – or -*I do not consent to participate in this survey*. If consent was declined, access to the online survey was terminated.

### Survey questionnaires

All survey questionnaires for studies 1–4 can be downloaded from Edge Hill University’s public data depository at https://doi.org/10.25416/edgehill.25260337.v1. Anonymised datasets for studies S1-S4 can be accessed through this public depository at https://doi.org/10.25416/edgehill.30002962.v1.

## Results

### 1. Learning during the pandemic & adjusting to a new challenging situation

This study captured students overall learning experiences after several months of restricted blended learning (November 2020), and then again in March 2022 and April 2023 as detailed in Table 3. Students were also invited to provide free-text feedback on their overall learning experiences.

**Table 3:** Overall learning experience. *Results shown in percentages.*

*“Overall learning experience”*	Study 1 (Nov 20)			Study 3 (Mar 22)			Study 4 (Apr 23)		
	L4 (26/26)	L5 (28/28)	L6 (16/16)	L4 (30/30)	L5 (59/59)	L6 (41/41)	L4 (27/27)	L5 (41/41)	L6 (15/15)
Excellent	11.5	3.6	25.0	13.3	10.2	12.2	3.4	10.0	21.4
Very Good	46.2	25.0	6.3	43.3	42.4	31.7	37.9	15.0	42.9
Good	26.9	32.1	56.3	33.3	40.7	46.3	34.5	40.0	14.3
Average	3.8	28.6	12.5	0.0	6.8	4.9	24.1	25.0	14.3
Poor	3.8	7.1	0.0	6.7	0.0	2.4	0.0	7.5	7.1
Very Poor	0.0	3.6	0.0	3.3	0.0	2.4	0.0	2.5	0.0
Prefer not to say	0.0	0.0	0.0	3.3	0.0	2.4	0.0	0.0	0.0
Overall satisfaction (Excellent – Good)	84.6	60.7	87.7	90.0	93.2	90.2	75.9	85.5	78.6

Explanation: Overall learning experience at the biology department 2020−2023 as experienced by students throughout the Covid-19 pandemic and in follow-up years. Numbers in brackets indicate the total participants followed by total answers provided. Overall satisfaction is the sum of results provided for rubrics “excellent”, “very good”, and “good”. Single-answer question. *Results displayed in percentages*

#### Academic year 2020–2021 (autumn semester/ study 1).

On September 22^nd^, 2020, the UK government announced a new work-from-home policy and introduced a second national lockdown on November 5^th^, 2020, followed by student-specific pre-Christmas travel window, enabling students to return to family and friends for the festivities. Covid-19 and frequent regulatory and legal updates resulted in short-notice timetable changes to students returning to academic year 2020–2021 [29–31].

Consequently, students in this study were taught in a blended approach from the start of the 2020/21 academic year with students attending on campus once a week for face-to-face sessions and practical activities, while being taught remotely all other days/sessions. Sessions on campus had been streamed live to enable an all-inclusive learning environment for those unable to attend on campus (shielding, travel restrictions).

A first-year biology student suggested this summary on their overall learning experience at the biology department, captured in Study S1 (November 2020):

*“As someone who has not been on campus due to Covid-19, it has been difficult to keep track of everything and maintain a routine. However, the tutors have been excellent and have worked very hard to ensure things are running smoothly” (L4/9452/5/S1).*

This contrasts with a comment made by a third-year genetics student in the same study, highlighting the technical and IT hurdles many students encountered whilst learning remotely:

*“Poor internet connection meant that my mum, brother, and I couldn’t work from home effectively at the same time” [….] (L6/9140/13/S1).*

Students shared their preferred location for attending remote and blended learning sessions during the autumn 2020 semester, while teaching at most English higher education institutions was restricted to an online or blended teaching manner with very limited access to on-campus facilities and laboratories. At this time bioscience students at our department studied in a remote fashion on 4 out of 5 days and attended on campus on one day per week with different year groups attending on different days to limit the spread of the Sars-Cov2 virus. Table 4 displays the preferred workplaces for students attending remote teaching in November 2020.

**Table 4:** Working at home – students’ workplaces. *Results displayed in percentages.*

“*Where do you learn and attend remote sessions?”*	Study 1 (Nov 20)		
	L4 (26/42)	L5 (28/61)	L6 (16/31)
A private room in the library	7.7	7.1	12.5
My bedroom/ my room in halls/ on campus	84.6	64.3	56.3
The kitchen table/ sharing a large desk with others	15.4	25.0	31.3
The dining room table	11.5	35.7	18.8
My bed	19.2	53.6	37.5
A coffee shop (around the corner)	3.8	0.0	6.3
My car	3.8	3.6	0.0
While at work	3.8	3.6	6.3
At my parents’	11.5	25.0	25.0

**Explanation:** Students at the Biology department described where they learned and attended remote and blended sessions in semester 1 of academic year 2020−2021 while local and national Covid-19 policies restricted on-campus activities, travel, and social life. This is a multiple answers question. Numbers in brackets indicate the total participants followed by total answers provided. Results displayed in percentages.

Students provided critical feedback on recorded online learning content intended for independent asynchronous study, predominantly made available prior to a scheduled class (Table 5). Recorded content was made available on the LMS (Learning Management System, Blackboard™) enabled access to all students with internet/IT access.

**Table 5:** Remote learning: Preferences on recorded content. *Results displayed in percentages.*

“*Which format and style of recorded content do you prefer?”*	Study 1 (Nov 20)		
	L4 (26/72)	L5 (28/58)	L6 (16/33)
Pre-recorded content/presentations are generally well made	61.5	60.7	68.8
Pre-recorded content/presentations are often too long (longer than 20 minutes)	42.3	17.9	6.3
I struggle with time management if presentations are too long	38.5	21.4	12.5
I am ok with longer pre-recorded content/presentations as long as I can stop/start to my needs	50.0	46.4	18.8
I am so happy we can now study (more) in our own time – this helps me with my own personal time management	34.6	39.3	43.8
Many pre-recorded content/presentations are of poor quality	7.7	0.0	0.0
I think pre-recorded content/presentations should not be longer than 5–10 minutes, followed by a quiz	15.4	7.1	18.8
I would prefer there is more advice on time-management on when and how to study pre-recorded material	26.9	14.3	37.5

**Explanation:** Students at the Biology department described their preferences for recorded content made available on the LMS for asynchronous learning prior to a scheduled session. This is a multiple answers question. Numbers in brackets indicate the total participants followed by total answers provided. Results displayed in percentages.

Participants provided 31 free-text responses (15, 11, and 5 responses at L4, L5, and L6, respectively) detailing their views on recorded content in Study S1 (November 2020): Participants preferred recordings which provided closed captures, and showed a clear structure (table of content, slide-by-slide structure). Participants voiced a preference for recordings and PowerPoint slides made available separately, enabling students to *read along* and make annotations while listening to the recordings.

With only limited on-campus sessions available for most of academic years 2019/20 and 2020/21 many students had reported a lack of structure and the wish for additional guidance for their remote learning routines, represented in a statement by a year 1 plant science student captured in Study S1 (November 2020):

“**[….] Clear, concise [work] instructions for everything” (L4/3902/*62/S1).*

A fellow first year student, studying towards a bachelor’s degree in biomedical science, summarised their feelings about new and unknown staff and the challenges of all-remote modules in a statement captured in November 2020:

*“I feel that having set modules which are taught exclusively online is negatively affecting my studies, I find it harder to understand and harder to ask for help having never met those [who] are teaching me, maybe at least a few sessions in each module could be taught face to face to assist [my] learning” (L4/6421/62).*

In November 2020 we asked students about their newly adopted learning, planning, and assessment preparation strategies as a consequence of the pandemic and the need to work from home (multiple-answer question, Table 6).

**Table 6:** Skills development and coping strategies as a consequence of the pandemic. *Results displayed in percentages.*

*[…] Below is a list of attributes and actions which might apply to yourself; please select those you have NEWLY adapted as a consequence of the blended learning style the university has now implemented.*	Study 1 (Nov 20)		
	L4 (26/77)	L5 (28/77)	L6 (16/42)
I have allocated a space in my room as my workspace	42.3	50.0	37.5
I have defined my own working hours and I do not work outside these working hours	23.1	25.0	25.0
I tell others (in my household) when I study and that I am not to be disturbed	38.5	50.0	31.3
I use ear plugs when I study (to exclude external noises) – My work is important	15.4	17.9	25.0
I have started an at-home exercise regime to keep myself fit	23.1	17.9	18.8
I have become more independent in my learning and more pro-active	42.3	17.9	12.5
I have learned to plan in a weekly manner	30.8	25.0	37.5
I am more pro-active in seeking support from various sources available from the university	19.2	21.4	37.5
I am now more confident in using software tools and remote technology	53.8	35.7	31.3
not applicable	7.7	10.7	6.3
prefer not to say	0.0	3.6	0.0

Explanation: Coping strategies developed and adopted by students to adapt to the new remote and blended working requirements as a consequence of the Covid-19 pandemic in autumn 2020. Multiple answers possible. Numbers in brackets indicate the total participants followed by total answers provided.

Survey participants voiced their opinion on how the department could assist students in their eLearning and might offer coping strategies in 38 free text responses (L4: 14, L5: 16, L6: 8) with 42.1% (16/38) responders expressing a wish for more non-modular and fun activities offered online, 34.2% (13/38) against, and 26.3% (10/38) outlining other/personal statements and feedback; for example a year 1 genetics student expressing their wish for more extra-modular activities:

“**Both during lectures and within separate bioscience group chats we only talk about academic topics and do not know each other well (especially those from other teaching groups). I find most of my social interaction/people I know best through societies, so maybe having optional fun sessions could help students interact with each other more” (L4/0711/5*7/S1).*

A year 2 biology student living in private accommodation in the nearby town of Ormskirk expressed a different opinion:

“**I live alone and the only time I talk to anyone since I’ve come back to Ormskirk is for a few minutes before I go into the [X] lecture on Thursday. I spend 24 hours of most days in a 4m x 4m room alone. I don’t really think extra sessions would make much of a difference for me personally” (L5/0378/57/*S1).*

For any on-campus teaching in semester 1 in academic year 2020/21, students were allocated into small groups (“bubbles”) allowing the required two meter distanced spacing during classroom teaching. Each student received their own set of equipment for any lab experiment. In the November 2020 survey (Study S1) students had been asked if they felt safe with the offered on-campus teaching arrangements; 88.5% of all L4 students (28 participants providing 28 responses) fully/to some extent, agreed that it was safe to be in a classroom, as well as 85.7% of all L5 students (26/26) and 62.7/% of L6 students (16/16).

Students acknowledged the extra workload that technical staff were experiencing as 92.3%, 85.8%, and 68.8% students L4-L6 fully or to some extent agreed that “*technical staff do an amazing job preparing each session*”. Students in levels L4-L6 agreed fully or to some extent with 96.2%, 96.4%, and 75%, respectively that “*all staff do they best they can, given the circumstances*”.

#### Academic year 2020–2021 (spring semester/ study 2).

Students and staff worked from home during the third [complete] national lockdown from January 6^th^ to March 7^th^, 2021, which also included the semester 1 exam period. Exams were conducted online as time-limited assessments (TLAs). Staff and year 2 students returned to campus for a (laboratory/fieldwork) research week on March 8^th^, 2021, while all other students continued to work from home until March 15^th^, 2021, when teaching resumed in a blended fashion as described above.

Study S2 was conducted in April 2021, a few weeks after students returned from the third national lockdown (January 6^th^ to March 7^th^) to blended but still restricted on-campus learning modalities. Table 7 depicts students’ viewpoints on the impact of the 3^rd^ national lockdown, and the consequential remote-only teaching on their learning.

**Table 7:** Effects of the third national lockdown on learning for bioscience students. *Results displayed in percentages.*

*“To which extent was your learning affected by the national lockdown in spring 2021”*	Study 2 (Apr 21)		
	L4 (16/16)	L5 (11/11)	L6 (7/7)
Absolutely	25.0	72.7	28.6
Partly	37.5	18.2	6.3
depends on session content	37.5	9.1	12.5
depends on module	0.0	0.0	12.5
not at all	0	0	0

Explanations: Students’ perceptions of the effects of the UK’s 3rd national lockdown (January to March 2021) on their learning at a Higher Education Institution (university). Results from the Learn!Bio study 2 in April 2021. Numbers in brackets indicate the total participants followed by total answers provided. This is a single-answer question. *Results displayed in percentages.*

In April 2021 this study enquired about changes to students’ accommodation provisions as a direct consequence of the Covid-19 pandemic, including campus closures, national lockdowns, or changes in their finances or personal circumstances. Table 8 outlines changes in accommodation(s) for bioscience students at Edge Hill University in academic year 2020–2021.

**Table 8:** Covid-19 pandemic and living arrangements for students. *Results displayed in percentages.*

*“Taken together, how often did you change accommodation this academic year? Please count only study-related changes of accommodation. “*	Study 2 (Apr 21)		
	L4 (16/16)	L5 (11/11)	L6 (7/7)
Never (0x)	56.3	45.5	14.3
Once (1x)	18.8	0.0	42.9
Twice (2x)	25.0	36.4	28.6
Three times (3x)	0.0	9.1	14.3
Four times (4x)	0.0	0.0	0.0
More than four times (> 4x)	0.0	9.1	0.0

**Explanation:** Changes in accommodation as a direct effect of the pandemic in academic year 2020–2021, as reported by bioscience students at the biology department. Numbers in brackets indicate the total participants followed by total answers provided. This is a single-answer question. **Results displayed in percentages. Students’ living arrangements in November 2020 are captured i*n* ***S1 Table*** *i*n the supplements.**

The national and local pandemic policies and regulations in academic years 2019/20 and 2020/21 imposed restrictions on lab-based and field studies. Working at home and attending live online, blended, and recorded sessions from home was an entirely new experience for most of our students during the pandemic. The learning from home experience added unexpected challenges to students’ education and learning, including technical, IT and connectivity issues and persisting struggles with personal well-being, anxiety, and depression, as summarised in Table 9.

**Table 9:** Challenges and hurdles experienced by bioscience students while working from home. *Results displayed in percentages.*

*“Working from home can be challenging […] Please tick of the below statements which describe your learning experience since the start of this semester.”*	Study 1 (Nov 20)			Study 2 (Apr 21)		
	L4 (26/60)	L5 (28/81)	L6 (16/43)	L4 (16/43)	L5 (11/49)	L6 (7/19)
Too loud (external noises, for example from street)	30.8	32.1	25.0	12.5	36.4	14.3
Too loud (from children or other household members, e.g., other students)	26.9	42.9	14.3	18.8	63.6	71.4
Not enough space (e.g., when living in halls)	23.1	28.6	14.3	6.3	18.2	14.3
Internet problems (connectivity is poorly)	38.5	60.7	35.7	37.5	72.7	42.9
No time (e.g., when needed to work or to care for children or the elderly)	15.4	28.6	10.7	18.8	36.4	0.0
Problems to concentrate in these settings	61.5	64.3	42.9	56.3	90.9	71.4
Equipment issues (not owning a personal computer/computer too old)/ I don’t own a computer	3.8	25.0	7.1	0.0	0.0	0.0
I have care responsibilities and I am constantly worried and overworked. I hardly sleep **(3)**	N/A	N/A	N/A	6.3	27.3	14.3
I cannot access the hardcopy books/literature I require. I do not own a printer/ I cannot access a printer **(3)**	N/A	N/A	N/A	18.8	36.4	14.3
I am suffering from depression/anxiety. The social distancing rules and insulation of the pandemic do not help either. This all is not aiding my learning process. **(3)**	N/A	N/A	N/A	37.5	63.6	28.6
Not applicable	30.8	7.1	3.6	6.3	9.1	14.3
Prefer not to say	0.0	0.0	0.0	6.3	9.1	14.3

**Explanation:** Examples of technical, practical, social, and mental well-being challenges student faced while working from home and attending remote sessions from home in academic year 2020–2021. Multiple answers possible. Numbers in brackets indicate the total participants followed by total answers provided. Results displayed in percentages. **(3)** These questions have not been asked in Study 1; (N/A) – data not available. Additional student feedback for study S2 is provided in S2 Fig.

### 2. Wellbeing & resilience

#### Academic year 2020–2021 (spring semester/ study 2).

**Fig 2:**
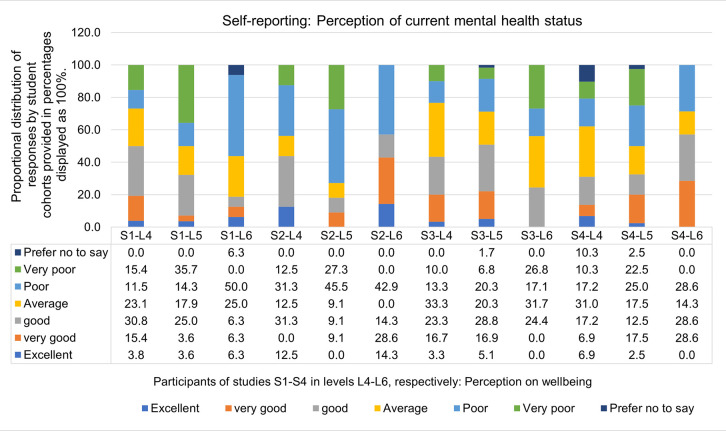
Participants perception of wellbeing. Participants perception of their own current wellbeing from November 2020 (Study 1) to May 2023 (Study 4). Students have been surveyed in November 2020 (S1-L4, S1-L5, S1-L6), April 2021 (S2-S4, S2-L5, S2-L6), March 2022 (S3-L4, S3-L4, S3-L6) and May 2023 (S4-L4, S4-L5, S4-L6). For cohort sizes and student grouping see Table 1. Single-answer question. Data shown and displayed in percentages.

The advent of the Covid-19 pandemic and the restrictions implemented on national and local levels resulted in extensive uncertainty amongst our students. The third national lockdown in spring 2021 (Jan 6^th^ – March 7^th^) resulted in an exponential decrease in students’ wellbeing (Study S2), with 43.7% of level 4 (L4) students, 72.5% of level 5 (L5), and 42.9% of level 6 (L6) students reporting of “poor” or “very poor” mental health (Fig 2), also reflected in Table 10. All year groups reported a “large” mental health decline from semester 1 to semester 2 in academic year 2020–2021 (study conducted shortly after the third national lockdown). Such a prominent change in wellbeing was not observed again during the lifetime of this study.

**Table 10:** How mental health changed compared to last semester. *Results displayed in percentages.*

Change in mental health past semester to this semester	Study 2 (Apr 21)			Study 3 (Mar 22)			Study 4 (Apr 23)		
	L4 (16/16)	L5 (11/11)	L6 (7/7)	L4 (30/30)	L5 (59/59)	L6 (41/41)	L4 (27/27)	L5 (41/41)	L6 (15/15)
large improvement	12.5	9.1	57.1	10.0	22.0	14.6	6.9	12.5	0.0
small improvement	6.3	9.1	14.3	0.0	0.0	0.0	20.7	17.5	21.4
unchanged	31.3	9.1	0.0	30.0	30.5	12.2	20.7	15.0	42.9
small decline	6.3	9.1	14.3	26.7	10.2	24.4	27.6	30.0	35.7
large decline	**25.0**	**45.5**	**28.6**	3.3	1.7	2.4	13.8	17.5	0.0
unsure	31.3	36.4	14.3	33.3	37.3	48.8	3.4	2.5	0.0
prefer not to say	6.3	9.1	14.3	0.0	0.0	0.0	6.9	5.0	0.0

Explanation: Participants’ perception of changes to their mental health in comparison to the previous semester. Single answer question. Numbers in brackets indicate the total participants followed by total answers provided. Results displayed in percentages. Statistical analysis over all year cohorts L4-L6 and all studies S2, S3, and S4, respectively showed evidence of significance for a large decline in mental health with p = 0.00983.

Students reported a range of variables which might have affected their wellbeing and mental health in academic year 2020−2021, most prominently the lack of opportunities for communication with others (students, adults, peers), and missing a community feeling, which was even more evident after the third national lockdown (Jan 6^th^ to March 7^th^, 2021). From spring 2021 onwards, students were more exposed to bereavements caused by the SARS-CoV-2 virus infections and their consequences within close family or friends (Table 11).

**Table 11:** Consequences of remote and blended learning on participants wellbeing in academic year 2020-2021. *Results displayed in percentages.*

*“Do you believe the new blended learning approach, with less on campus and more remote teaching has an impact on your mental wellbeing? Please tick all statements which apply to your situation.”*	Study 1 (Nov 20)			Study 2 (Apr 21)		
	L4 (26/59)	L5 (28/108)	L6 (16/56)	L4 (16/50)	L5 (11/56)	L6 (7/19)
I feel lonely/more lonely	11.5	46.4	50.0	31.3	54.5	28.6
I miss a community feeling	38.5	42.9	43.8	50.0	54.5	42.9
I miss a feeling of belonging	7.7	35.7	31.3	37.5	45.5	71.4
I miss talking to people	38.5	64.3	62.5	50.0	54.5	42.9
I miss speaking to adults	7.7	32.1	12.5	18.8	45.5	14.3
I miss to relax/chill out with others	30.8	46.4	56.3	25.0	63.6	28.6
I feel depressed/anxious	11.5	32.1	18.8	31.3	81.8	14.3
I am anxious about the future/my studies	26.9	64.3	56.3	(#)	(#)	(#)
I am suffering from severe anxiety I am suffering from a clinically proven depression	(*)	(*)	(*)	50.0	63.6	28.6
There have been one or more bereavements affecting me/my family/my close friends	(*)	(*)	(*)	18.8	54.5	14.3

**Explanation:** Multiple answers possible. Numbers in brackets indicate the total participants followed by total answers provided. Results displayed in percentages. **(#)** question not asked in Study 2 (April 21), (*) question not asked in Study 1 (November 20).

During times of remote or blended learning students were supported by their respective year coordinator, personal tutors, and module leaders at the biology department, who all reached out to students on a regular basis. Frequent changes in national and local Covid-19 policies and regulations resulted in ad hoc timetable adjustments with often dire consequences for students’ learning experiences. In the April 2021 Study S2 feedback was sought on participants’ achievements on building-up resilience in these challenging times and how students perceived their own resilience at this stage (Table 12). Additional supportive information on resilience development can be found in supplemental S2 Table.

**Table 12:** Participants pathway to resilience at the height of the Covid-19 pandemic. *Results displayed in percentages.*

*“This question is about resilience. Below are a few statements. Read them carefully and then decide how you feel about each of these statements. When considering your answers, look back at the whole past academic year and how much you have developed. “*	Study 2 (Apr 21)		
	L4 (16/98)	L5 (11/43)	L6 (7/49)
Looking back at the past year, I have gained more confidence.	56.3	18.2	42.9
I am now more independent in my work	75.0	27.3	71.4
I am proud about what I have achieved.	62.5	54.5	85.7
I like who I am	50.0	27.3	85.7
I can do many things on my own, I can work in a self-paced manner.	62.5	54.5	85.7
I can set myself a goal and I can reach it	62.5	36.4	71.4
I can achieve high aims, something I have never believed I could do	18.8	9.1	42.9
I struggled with technology, but now I am good with all sorts of new technology	12.5	45.5	14.3
I study because I have a career plan. I study for myself, and this drives my day, every day.	37.5	27.3	14.3
Firstly, I was upset about the frequent timetable changes but now I have learned to take them as they come.	31.3	27.3	0.0
I am now more independent in my learning. I believe this will help me later in my career	62.5	18.2	85.7
If my family/my friends/my partner is around me, I feel totally in control of my life	37.5	27.3	42.9
I cannot control the virus, but I can work very hard to have high marks	43.8	18.2	57.1

Explanations: Responses from the *Learn!Bio* Study S2 in April 2021, one month after the third national lockdown had come to an end. Multiple answers possible. Numbers in brackets indicate the total participants followed by total answers provided. Results displayed in percentages.

### 3. Consequences of the pandemic & students’ expectations from providers

We conducted two post-pandemic follow-up surveys shedding light on long-term consequences for higher education bioscience students on learning experiences and wellbeing because of the Covid-19 pandemic. The first post-pandemic survey took place in March 2022 (Study S3) followed by a final survey in April 2023 (Study S4).

#### Academic year 2021–2022 (spring semester/ study 3).

Participants provided feedback on the (long-term) effects of (pandemic) online and blended teaching on their current learning progression (Table 13).

**Table 13:** Long-term effects of remote learning on current bioscience students. Results displayed in percentages.

*“Do you feel the past remote and blended learning during the pandemic affects your current learning?”*	Study 3 (Mar 22)		
	L4 (30/30)	L5 (59/59)	L6 (41/41)
Absolutely	16.7	35.6	41.5
Partly	20.0	23.7	19.5
Depends on session content	26.7	16.9	17.1
Depends on module	13.3	11.9	12.2
Not at all	23.3	10.2	9.8

Explanation: Consequences of online and remote teaching during the Covid-19 pandemic on current learning progression as perceived by students at the biology department at Edge Hill University captured at the post-pandemic follow-up survey in 2022 (Study S3). Single answer questions. Numbers in brackets indicate the total participants followed by total answers provided. Results displayed in percentages.

#### Academic years 2022–2023 (spring semester/ study 4).

In April 2023 (Study S4) participants provided insights to which extend the consequences of the pandemics still affected their [current] social life (Table 14).

**Table 14:** Social life consequences of the Covid-19 pandemic. *Results displayed in percentages.*

*“Has the pandemic affected your social life?”*	Study 4 (Apr 23)		
	L4 (30/30)	L5 (59/59)	L6 (41/41)
Yes, by a large amount	**31.0**	**30.0**	**42.9**
yes, by a small amount	**27.6**	**30.0**	**57.1**
no change	**37.9**	**27.5**	0.0
unsure	3.4	12.5	0.0

**Explanation:** Long-term effects of the Covid-19 pandemic on bioscience students’ social life surveyed in April 2023. Single answer questions. Numbers indicate the total participants followed by total answers provided. Results displayed in percentages.

Amongst all participants surveyed in this study at level L4, 56% report about anxiety (disorders) and/or autism spectrum disorders (ASD), as well as 38% at level L5, and 42% at level L6, respectively. A year 2 biotechnology student expressed their struggles with on-campus learning in this statement, captured in the Study S3 (March 2022):

*“[…] The long drive to uni[versity] affects my ability to learn at in-person lectures - I feel, much like last year, I would learn a lot more sat at home in my own environment. […] As someone with autism it’s difficult to form new routines and sometimes, as silly as this sounds, my entire day can be ruined from just one thing like someone sitting in a seat I usually sit in which is why it’s important to ensure that online learning is still an option when it comes to lectures as, with some of us, the commute can seriously impact our ability to learn” (L5/5418/26a/S3).*

As a result of the Study 3 preliminary data analysis (Table 13) and conversations with students we extended our questionnaire for the final survey in April 2023 allowing additional insight on participants’ pre-university learning restrictions during the Covid-19 pandemic (Tables 14 and 15).

**Table 15:** Effects of students pre-University learning on current university progression. *Results displayed in percentages.*

*“Covid-19 and the pandemic: Has your pre-Uni and early-university learning had an impact on your current studies?”*	Study 4 (Apr 23)		
	L4 (27/27)	L5 (41/41)	L6 (15/15)
Absolutely	27.6	40.0	28.6
Partially	27.6	40.0	28.6
Not at all	34.5	15.0	28.6
Unsure	3.4	2.5	14.3
Prefer not to say	6.9	2.5	0.0

**Explanation:** Single answer questions. Numbers in brackets indicate the total participants followed by total answers provided. Results displayed in percentages.

**Table 16:** Level of learning affected by Covid-19. *Results displayed in percentages.*

*“What level of learning pre-University as well as which years of your learning at university have been affected by Covid-19 restrictions?”*	Study 4 (Apr 23)		
	L4 (28/48)	L5 (41/57)	L6 (14/29)
GSCEs	48.3	2.5	0.0
BTEC	24.1	17.5	14.3
AS-levels	13.8	30.0	0.0
A-levels	62.1	52.5	71.4
L4 (year 1) at university	3.4	25.0	85.7
L5 (year 2) at university	0.0	5.0	21.4

Explanation: Students’ learning affected by Covid-19 at university, schools, and colleges: Students surveyed might have been affected by the pandemic prior to attending university and at various stages during their education. Multiple answers possible. Numbers in brackets indicate the total participants followed by total answers provided. Results displayed in percentages.

### Years after the pandemic – studies S3 and S4 compared

In the 2022 follow-up study (Study S3) students still felt the imminent effects of the pandemic and voiced their estrangement from a pre-pandemic social behaviour, which was less evident in the 2023, Study 4 (Table 17).

**Table 17:** Personal and social attributes and how these inform student’s perception of post-pandemic teaching and learning. *Results displayed in percentages.*

*“Reflect on your approach to teaching and learning this academic year.”*	Study 3 (Mar 22)			Study 4 (Apr 23)		
	L4 (30/79)	L5 (59/178)	L6 (41/119)	L4 (27/44)	L5 (41/63)	L6 (15/24)
I am excited to be getting back to pre-pandemic learning	50.0	54.2	53.7	3.7	24.4	40.0
I am anxious about being in contact with large number of people during a pandemic	30.0	27.1	14.6	14.8	12.2	6.7
I am l excited to meet my class/roommates in person	36.7	39.0	56.1	18.5	9.8	13.3
I am anxious about the lack of social distancing/mask wearing on campus/my commute	10.0	15.3	4.9	7.4	4.9	6.7
I watched videos during remote learning to gain practical knowledge, now I feel I am prepared to learn and develop those lab skills.	10.0	13.6	4.9	7.4	7.3	6.7
**I am anxious about my lack of practical knowledge from missed in-person teaching**	**30.0**	**52.5**	**46.3**	**25.9**	**24.4**	**26.7**
The lockdowns gave me a nice break from the everyday social situations	**53.3**	**64.4**	**61.0**	14.8	22.0	20.0
The lockdowns have made me more self-conscious in everyday social situations	30.0	33.9	43.9	37.0	17.1	26.7
N/A – There has been no impact on my learning	13.3	1.7	4.9	18.5	26.8	0.0

**Explanation:** Multiple answers possible. Numbers in brackets indicate the total participants followed by total answers provided. Results displayed in percentages.

Throughout the pandemic, students acquired various new IT and computer skills. In our follow-up surveys in 2022 and 2023, respectively, we sought feedback from participants how they felt about their IT literacy and technology advancements (Table 18).

**Table 18:** IT technology & the post-pandemic generation of HE learners. *Results displayed in percentages.*

Do you feel you are now more technology advanced?	Study 3 (Mar 22)			Study 4 (Apr 23)		
	L4 (30/30)	L5 (59/59)	L6 (41/41)	L4 (27/27)	L5 (41/41)	L6 (15/15)
Yes	**86.7**	**81.4**	**75.6**	**85.7**	**87.8**	**92.9**
No	10.0	16.9	24.4	10.7	0.0	0.0
Unsure	n.a.	n.a.	n.a.	7.1	9.8	7.1
Prefer not to say	3.3	1.7	0.0	n.a.	n.a.	n.a.

**Explanation:** Technology advanced learners. Participants reflection on their ability to use computers. and online technology as compared to before the pandemic with remote and blended learning requiring them to embrace these new tools and technology. (n.a.) – not asked. Single answer questions. Numbers in brackets indicate the total participants followed by total answers provided. Results displayed in percentages.

Students in this study returned to pre-pandemic, on-campus learning setting from September 2021, as requested by the Office for Students (OfS) and enabled by national Covid-19 regulations [32]. With digital teaching technology now widely available enabling colleagues to deliver blended and online sessions, students’ perceptions on future teaching practices were captured in Study 3 (2022) and Study 4 (2023). Students expressed a clear preference for a combination on blended and face-to-face teaching but rated purely online teaching less favourably (Table 19). Preferences had not changed from 2022 to 2023.

**Table 19:** Students preferences on teaching post-pandemic: *Results displayed in percentages.*

*“Please indicate your preferred teaching and learning style at university”*	Study 3 (Mar 22)			Study 4 (Apr 23)		
	L4 (30/30)	L5 (59/59)	L6 (41/41)	L4 (28/28)	L5 (41/41)	L6 (14/14)
Online learning (off campus)	0.0	1.7	0.0	10.3	2.5	0.0
**blended learning**	**56.7**	**49.2**	**41.5**	**44.8**	**42.5**	**50.0**
**face-to-face (on campus)**	**36.7**	**47.5**	**58.5**	**44.8**	**55.0**	**50.0**
no preference	6.7	1.7	0.0	0.0	0.0	0.0

**Explanation:** Bioscience students’ preferences for online, blended, and face-to-face (on campus) teaching as identified in the S3 and S4 surveys, respectively. Single answer questions. Numbers in brackets indicate the total participants followed by total answers provided. Results displayed in percentages.

Students were surveyed about which of the non-LMS embedded learning tools and software introduced for remote and blended learning during the Covid-19 pandemic, that the department should continue to use in its teaching delivery. Students from all cohorts mentioned YouTube (54.7%, range 48.8–65.5%), Kahoot! (49.4%, range 39.9–52.5%), LinkedIn Learning (8.9%, range 3.3–26.8%), Socrative (11.5%, range 3.3–20.3%), Padlet (10.1%, range 0.0–29.3%), and Clinical Key Students (12.3%, range 3.3–26.8%). Not all students in all year groups had previous experience with all mentioned online learning tools.

We captured our final year undergraduate bioscience students’ career plans and how these have been impacted by the Covid-19 pandemic and its consequences on access to training, job-preparedness, and availability of graduate bioscience roles (Table 20). During the pandemic we observed that a higher than usual number of students continued with their education (MRes, MSc, PhD), either with us or elsewhere instead of pursuing a paid-for career. A year 3 (L6) ecology & conservation student’s voice from the November 2020 (Study S1) summarises this reasoning:

**Table 20:** Bioscience students career plans during and after the Covid-19 pandemic. *Results displayed in percentages.*

*Career plans for bioscience graduates*	Study 1 (Nov 20)	Study 2 (Apr 21)	Study 3 (Mar 22)	Study 4 (Apr 23)
	L6 (16/26)	L6 (7/7)	L6 (41/41)	L6 (15/13)
**My post-graduation plans have not changed**	**26.9**	**57.1**	**36.6**	**46.2**
I never had any post-graduation plans	0.0	0.0	12.2	15.4
I am now unsure about my future	15.4	28.6	17.1	15.4
My plans have now changed	19.2	0.0	**29.3**	15.4
**I will just wait how things develop and then decide**	**34.6**	**14.3**	(*)	(*)
There aren’t any jobs for what I want to do	0.0	0.0	0.0	7.7
With the pandemic there are now more jobs in my dream role than ever	3.8	0.0	2.4	0.0
I prefer not to say	3.8	0.0	2.4	0.0

**Explanation:** Career plans throughout and post-Covid-19. How bioscience undergraduate students’ career plans have been affected by the Covid-19 pandemic. Responses from final year (level 6) students surveyed in spring (March-May) of their final year. For studies S2-S3 single answer question survey, for S1 multi-answer survey. Numbers in brackets indicate the total participants followed by total answers provided. Results displayed in percentages.

“[The] **change of dissertation topic meant I had interest in a different postgraduate course (…), also I feel by doing a master [degree] I avoid the current situation with a lack of jobs (L6/0169/61*/S1)*”.

A human biology student in their final year expressed their worries about lack of job vacancies for bioscience graduates in the March 2021 Study S2:

*“I am applying for full-time jobs within the NHS, Civil Service, local universities, and other public services. I will continue working my part-time job until I secure a full-time position. […] I am concerned about high levels of unemployment at the minute making securing a job very difficult. I have already applied to a large number of jobs that I am sufficiently qualified for and have been rejected by every one of them. I think it is going to be a long process” (L6/2144/69/S2).*

## Discussion

### 1. Learning during the pandemic & adjusting to a new challenging situation

This manuscript evaluated students’ perception on studying and completing a bioscience degree during and after the Covid-19 pandemic from November 2020 to March 2023 in a cross-sectional, mixed-method approach by inviting undergraduate students to four independent, anonymous JISC online surveys as part of our departmental *Learn!Bio* study.

We firstly evaluated bioscience students’ perceptions on their learning experiences and how students adjusted to the practical and learning-relevant challenges which had arisen in the wake of the Covid-19 pandemic from November 2020 to spring 2021. All undergraduate students enrolled at the biology department at EHU were eligible to participate. Students were invited via a mass email sent to their student email inbox, which included a link to the anonymous JISC online survey. Students received a reminder email two weeks later.

Edge Hill University is a located in West Lancashire with a predominantly White (English, Welsh, Scottish, Northern Irish or British) population (96.9%, 2021 census [33]) population. Asian, Asian British or Asian Welsh citizens and Black, Black British, Black Welsh, Caribbean, or African citizens account for only 1.05% and 0.33% of the local community (2021 census [33]), respectively. While the university population is slightly more diverse, it still reflects the local demographic trends having a primarily White (92%) student body, with Asian (3%), Black (2%), Mixed (2%), and other ethnicities (1%) making up the remainder [34]; However, while 4.3% of the local population are non-UK passport holders (in comparison: Manchester: 19.2%, City of London: 34% [35]) only 2% of EHU student cohort are non-UK domiciled. Gender distribution at the university leans towards female students (70%), with males making up 30% and a significant portion of the student population is below 21 years old (75%), while 16% are aged 21–30, and 8% are over 30; However, all Biology students are aged 18 and above [34].

There is a significant internal disparity in income distribution in West Lancashire, with 15 out of 273 neighbourhoods ranked amongst the 20% most deprived with an income deprivation of 39%. In contrast 11/273 neighbourhoods in West Lancashire are ranked amongst the least deprived in England with an income deprivation of 2.7% [36]. This is also reflected in the university’s student cohort, with indices of multiple deprivation (IMD) data reveal that 15% of students are from the most deprived quintile (Q1), 48% fall into quintiles 2 and 3, and 38% are from the least deprived quintiles (Q4 and Q5) [36]. The university attracts a high number of local students who continue to live at home during their studies, the demographic data underscores the internal disparities in income distribution within this region as an example across the UK and highlights the essential role of all HEIs globally fostering an inclusive and supportive environment for students from varied backgrounds enabling access to higher education and academic opportunities.

Study satisfaction captured amongst the students in the November 2020 (Study S1, Table 3) was well above the national average, when compared with data from a national-wide Student Covid Insights Survey (SCIS) collected in October and November 2020 showing a 29% rate of dissatisfaction or severe dissatisfaction amongst surveyed students with their studies (71% satisfaction rate [13]). This contrasts with 88.1% and above of satisfaction amongst students in this study.

Staff at our university offered a mixture of synchronous and asynchronous teaching approaches with pre-recorded podcasts and presentations made available in preparation for on-campus or remote synchronous session. Most students agreed that remotely accessible learning content was generally well made (Table 5), a viewpoint also observed by other studies in the UK and globally [37,38]. Students in year 1 (L4) had only little opportunity to acquire the endurance and attention span to engage with lengthy learning content during their often-interrupted school/college education, which might explain their reluctance to engage with longer (20 minutes-plus) remote learning units [39].

Most students in this study felt they had been affected in their learning by the national lockdown and the online-only teaching in spring 2021 (Table 7). This result is supported by national-wide evidence from the 2021 National Student Survey (NSS) showing that only 47.6% of all respondents had been content with the delivery of their learning and teaching during the Covid pandemic, which was further emphasised by data made available from the Student Covid-19 Insights Survey (SCIS), reporting that 52% of students who had been in higher education during the pandemic felt that Covid-19 had a “significant” or “major impact” on their academic performance [13,40,41].

An above national average number of participants captured in this manuscript changed accommodation at least once during academic year 2020/21 (Table 8), with many students moving between their “home” and “study accommodation”. Nation-wide 81% of all students remained in their study-related accommodation in academic year 2020/21 [40].

A high percentage of students in this cohort lived off campus, predominantly with their parents (43%) or with partners/with their own children (18%) during the height of the pandemic, a factor which might has decreased their satisfaction with their studies, as space and IT resources for learning might have been restricted. A recent study by colleagues from the Aston University has shown that students from more deprived households had rated their study experiences less favourable during the pandemic, as these students had more frequently been deprived in one or more dimension [37], and might have lacked access to their own computer, fast internet or a suitable learning environment.

Accessing timetabled eLearning sessions with a lack of technology and/or a quiet space to concentrate was identified as a main hurdle for many participants as captured in November 2020 (Study S1). Access to a personal computer, computer accessories (microphone, camera) and internet connectivity was also a worry for many participants (Table 9). Our university, as many other HEIs in the UK, provided students in need, with laptops and associated equipment (cameras, headsets etc) for long-term loan, along with internet dongles to enable equal access to eLearning for all students. This resulted in the eradication of unobtainable access to personal computers by March 2021 (Table 9).

### 2. Wellbeing & resilience

An NHS digital survey analysis conducted on members of the public living in England and published in 2016 concluded that one in five women and one in eight men suffered from a common mental health condition prior to the Covid-19 pandemic [42]. Anxiety and symptoms of anxiety had been more evident in the UK general population before the Covid-19 pandemic (21%) compared to other countries, for instance Belgium (11%), New Zealand (6.1%), the USA (8.2%) or Sweden (14.2%), as recently reported by The King’s Fund (see reference [43], pages 33 and following). The marked differences in levels of anxiety and related conditions experienced by members of the public in the UK as compared to members of the public in other countries increased during the Covid-19 pandemic, with only 30.8% of the public in the USA expressing symptoms of anxiety, and 22.3% of the public in EU member states (averaged) reporting of anxiety symptoms during these times. This compares to 49.6% of the public in the UK with anxiety symptoms during the pandemic [43,44].

The UK experienced a full, strict national Covid-19 lockdown from January 6^th^ to March 7^th^, 2021.

Shortly after the third national lockdown, in April 2021, data for the second survey (Study S2) was captured, which showed a marked decrease in students overall wellbeing (Fig 2). Students at level 5 (class of 2019−2022, shown in yellow), reporting well below the national average on poor mental health as compared to all UK adults. This student cohort’s mental health, captured at this time point was also well below the reported average as reported in the April Student Covid-19 Insights Survey, in which 63% of all students expressed”*slightly or much worse now*” mental health since their started their studies in autumn 2020 [40] This trend in mental health decline amongst university students was also identified in a recent study by Chen and Lucock [45].

Students of the class 2019–2022 (displayed with yellow background) had overall reported more critical feedback and negative impressions in all surveys S1-S3. The class of 2019–2022 experienced Covid-19 restrictions throughout all years of their undergraduate studies, from the spring semester 2020 to summer 2022. This was also evidenced in students’ perception on their mental health changes from autumn 2021 to spring 2022 (Table 10), with students in the cohort of 2019–2022, showing a more severe decline in their wellbeing compared to students in the class of 2020–2023 cohort (displayed with green background). Nation-wide, students’ mental health experienced a further decline from autumn 2021 to spring 2022, as evidenced in the SCIC March 2022 survey [46].

Recent studies have shown that restrictions to campus life and student services affected those students from disadvantaged socioeconomic backgrounds, with often poor “*networking capital*” [12] and learners with (child) care responsibilities or inadequate IT equipment or internet connectivity, more severely [45,47]. Interestingly, our data indicates that campus closures and restrictions to libraries, IT rooms and on-campus teaching had a cross-sectional impact on all students in all years, except for the 2020−23 cohort (shown with green background) captured in the November 2020 study (S1), who might have capitalised on their previous (school/college) social network structures for support and wellbeing (Table 11) during their first term at university.

In the April 2021 survey (S2) we captured students’ perception on the development of new skills and students’ pathway to resilience (Table 12, supplementary Table S2). Feedback from students on their resilience and acquired skills seems to conflict with an overall perception of their current wellbeing at this time (“poor”, “very poor”), as students in years 1 and 3 (levels 4 and 6) provided a positive self-rating for their transition to resilience and have recognised the acquisition of various new soft skills, IT skills, as well as job-relevant graduate attributes required for a role in biosciences. Students in year 2 (level 5) had been more critical about their skills development and resilience advancements while these students expressed the largest decline in mental health across all year groups (45.5%).

### 3. Consequences of the pandemic & students’ expectations from providers

In two follow-up, post-pandemic surveys this manuscript captured students’ perception on long-term consequences of blended and remote learning on their bioscience education, wellbeing, and social life in academic years 2021−2022 (Study S3) and academic year 2022−2023 (Study S4). Responses show that 76.7%, 88.1%, and 90.2% of all students in years 1, 2, and 3, respectively, indicated that the previously experienced Covid-19 pandemic restrictions affected their higher education in 2022 (Table 13). Interestingly this effect continued to impact 51.9%, 56.1%, and 86.7%, of all year 1, 2, and 3 students, respectively, one year later (Study S4, Table 15).

Covid-19 had impacted not only students’ higher education but also their school and college education (Table 16), with those from the most deprived backgrounds reporting more often that they have fallen behind (42%) than those from the least deprived backgrounds (26%) [48]. Many if not all outreach activities had been cancelled during the pandemic years, affecting career choices and accession to university programmes, with those from deprived areas more affected than others [47]. Our findings are supported by a recent meta-analysis investigating student learning outcome across various countries by De Pietro *et al*. concluding with a significant learning deficit in math and science, as a consequence of pandemic learning, indicating that long-term support in STEM subjects might be required for school and early-stage university students, allowing equal and fair chances for all learners catching up with their learning [49].

In our study, students’ perception of their IT and computer literacy had notably increased from November 2020 to May 2023 with 92.9% of the students in the 2020–2023 cohort (green) self-reporting on improved IT skills (up from 12.5%, Table 12 vs Table 18), which is indicative of a pathway to resilience and an adaption to a new learning environment. This is further evidenced by feedback provided to the question on how future teaching should be mediated (online, blended, campus-based/face-to-face). Students were supportive of both, face-to-face and blended synchronous teaching, with blended/hybrid teaching allowing students to attend live streamed sessions remotely (Table 19). Our department, as most HEIs in the UK require students to attend physically for all scheduled sessions. In various free text responses students reported their reasons not to attend on campus, including public transport not being available at all/on certain days/impacted by strike actions, care responsibilities, and sickness (including mental health and chronic conditions). Many students expressed worries about the increased living costs impacting their travel arrangements to/from campus for “*just this session*”.

The financial aspects of attending university sessions was also captured in the recent ONS Student Cost of Living Insights Study (SCoLIS) from spring 2023, which suggested that 91% of all students worry/worry very about their finances and that 49% of all students have financial difficulties [50]. A recent study by Salem and colleagues points to evidence on best learning outcomes with blended learning strategies while critically arguing for highest students’ overall motivation and satisfaction through face-to-face learning modalities [51].

The Covid-19 pandemic had increased the prevalence of anxiety and depressive disorders amongst all students in the UK and had made it difficult for those with autism spectrum disorders (ASD) and other common mental health disorders affecting their mental wellbeing in the return to face-to-face learning and on-campus life [15].

Interestingly, 53.3%, 64.4%, and 61% of all participants in year 1, 2, and 3, respectively, reported in the March 2022 (Study S3) survey that the “*lockdowns gave me a nice break from the everyday social situations*”, returning to 14.8%, 22%, and 20% for students in years 1, 2, and 3, respectively in the May 2023 (Study S4) survey (Table 17), indicating that most students had been impacted by the social restrictions on social distancing during the Covid-19 pandemic.

We asked year 3 (level 6) students about their postgraduation career plans as bio-scientists and if these plans have changed because of the pandemic (Table 20); this data should be studied with caution as feedback was provided by different student cohorts at different time points and at different cross points during and after the pandemic. Final year students of the class 2019–2022 (yellow) showed the highest level of insecurity in reference to their postgraduation plans, while this cohort also struggled most with their pathway to resilience and adaptability to a new learning environment.

## Conclusion

This study highlights the strengths of bioscience students from all backgrounds to complete an undergraduate degree while acquiring new, graduate-ready attributes and skills, including extended IT competencies during the Covid-19 pandemic. Our results have shown that students can report poor mental health while already developing resilience, indicating tailored support can aid students’ resilience performance. Students surveyed during the Covid-19 pandemic had adjusted with ease to digital teaching provisions with current students voicing a clear preference for a subject-specific approach to teaching post-pandemic, entailing a mix of blended (synchronous) and face-to-face (on-campus) sessions, thus reducing students’ significantly risen living costs and meeting students’ often complex personal circumstances.

### Study limitations

The limitations of the study are mainly focused on the characteristics of the sample group and the data collected. The study had a low participation rate, with a total of 317 out of 1220 (25.98%) voluntarily participants, all studying towards a BSc (Hons) degree in the biological sciences. Therefore, these results only represent the experiences of a small cohort of students all within in a similar discipline, making it difficult to generalise the results especially across different disciplines who may have a lesser dependency of practical skills. Most participants, by nature of the EHU student demographic, originated from the local area and were therefore subject to the disparity of pandemic experiences between some of England’s most affluent and deprived backgrounds as well as different lockdown restrictions. This resulted in increasing response bias due to some students experiencing poor internet/technological access (prior to March 2021), suitable study space, and/or caring responsibilities, along with differences in local infection peaks and various local authority lockdown enforcements. For example, some students or their family members may have been symptomatic and/or under more severe lockdown restrictions during the survey period leading to more negative responses. Those who may have stronger opinions and/or more free time would be more likely to respond and to make more thoughtful responses. Additionally, survey questions were subjective and open to wide ranging inter-individual interpretations, expectations and biases, i.e., level and changes of mood. The original study design aimed for a comprehensive understanding of student wellbeing, changes to learning and resilience during the Covid-19 pandemic with a focus on open-question answers and an invitation to share lived experiences, resulting in limitations to rigorous statistical analysis. The study captured students’ perception of wellbeing, learning experiences and transition to resilience across multiple timepoints, but we have not been able to follow-up on individual students, as this study was entirely anonymous; Furthermore we did not receive permission to integrate personalised demographic data, which hinders comprehensive multivariant data analysis.

## Supporting information

S1 Fig Learn!Bio study design flowchart. This study is based on a constructivism-based teaching, learning, and research worldview. Data collection was completed in a convergent parallel mixed method approach from November 2020 to April 2023.
(PDF)

S2 Fig Lack of microphone and camera use during online classes. Students’ responses to a multiple-answers question in the April 2021 study S2. For year 1 (level 4, L4), 16 participants provided 32 answers, for level 5 (L5) 11 participants provided 32 answers, and for level 6 (L6), 7 participants provided 18 answers. Results displayed in percentages.
(PDF)

S1 Table Students’ accommodations in semester 1, academic year 20/21. Responses from the *Learn!Bio* Study 2 in April 2021, one month after the third national lockdown had come to an end. Multiple answers possible. Numbers in brackets after the level L4-L6 indicate the total participants followed by total answers provided. Results displayed in percentages.
(PDF)

S2 Table New skills and talents developed during the Covid-19 pandemic. Responses from the *Learn!Bio* Study 2 in April 2021, one month after the third national lockdown had come to an end. Multiple answers possible. Numbers in brackets indicate the total participants followed by total answers provided. Results displayed in percentages.
(PDF)
