# Supplementary material for: Learn!Bio—A time-limited cross-sectional study on biosciences students’ pathway to resilience during and post the Covid-19 pandemic at a UK university from 2020–2023 and insights into future teaching approaches
Source: PLoS One. 2025 Sep 25;20(9):e0300824. doi: 10.1371/journal.pone.0300824 (PMC12463289; doi:10.1371/journal.pone.0300824)
Supplement: S1 Fig — This study is based on a constructivism-based teaching, learning, and research worldview. Data collection was completed in a convergent parallel mixed method approach from November 2020 to April 2023. (PDF) [file pone.0300824.s001.pdf]

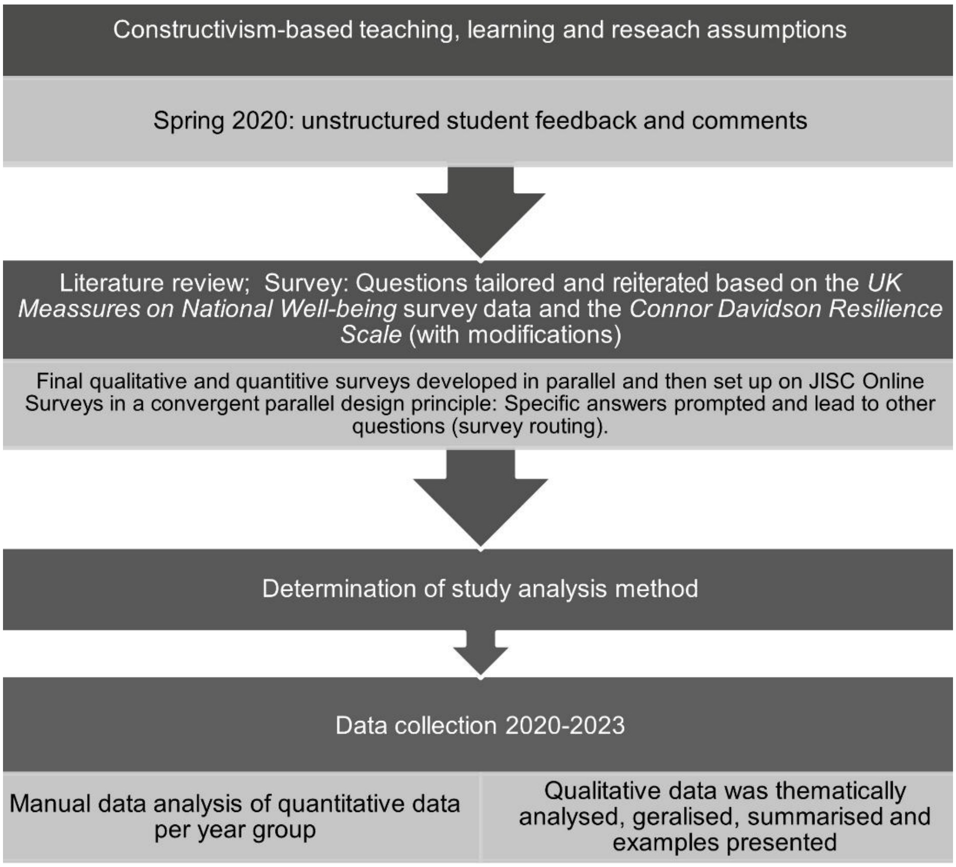

Figure S1: Learn!Bio study design flowchart. This study is based on a constructivism-based teaching, learning, and research worldview. Data collection was completed in a convergent parallel mixed method approach from November 2020 to April 2023.
