# Supplementary material for: Learn!Bio—A time-limited cross-sectional study on biosciences students’ pathway to resilience during and post the Covid-19 pandemic at a UK university from 2020–2023 and insights into future teaching approaches
Source: PLoS One. 2025 Sep 25;20(9):e0300824. doi: 10.1371/journal.pone.0300824 (PMC12463289; doi:10.1371/journal.pone.0300824)
Supplement: S2 Fig — Students’ responses to a multiple-answers question in the April 2021 study S2. For year 1 (level 4, L4), 16 participants provided 32 answers, for level 5 (L5) 11 participants provided 32 answers, and for level 6 (L6), 7 participants provided 18 answers. Results displayed in percentages. (PDF) [file pone.0300824.s002.pdf]

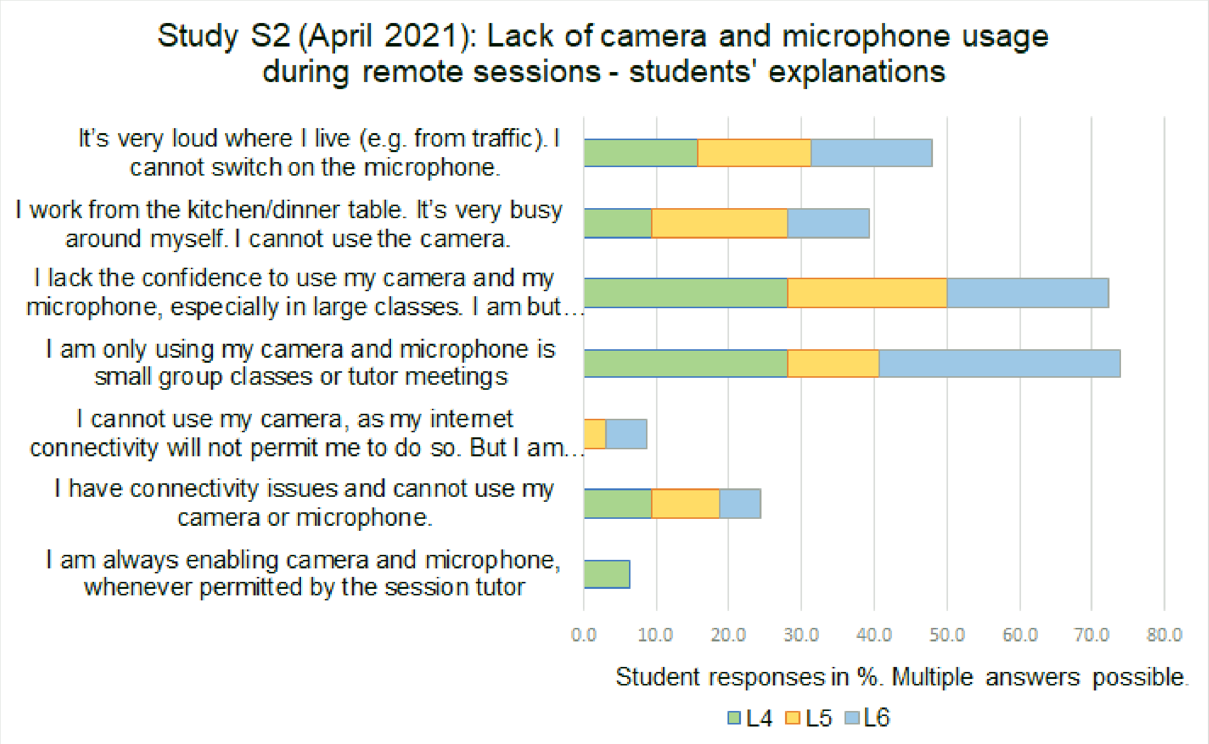

Figure S3: Lack of microphone and camera use during online classes. Students' responses to a multiple-answers question in the April 2021 study S2. For year 1 (level 4, L4), 16 participants provided 32 answers, for level 5 (L5) 11 participants provided 32 answers, and for level 6 (L6), 7 participants provided 18 answers. Results displayed in percentages.
