# Supplementary material for: Learn!Bio—A time-limited cross-sectional study on biosciences students’ pathway to resilience during and post the Covid-19 pandemic at a UK university from 2020–2023 and insights into future teaching approaches
Source: PLoS One. 2025 Sep 25;20(9):e0300824. doi: 10.1371/journal.pone.0300824 (PMC12463289; doi:10.1371/journal.pone.0300824)
Supplement: S1 Table — Responses from the Learn!Bio Study 2 in April 2021, one month after the third national lockdown had come to an end. Multiple answers possible. Numbers in brackets after the level L4-L6 indicate the total participants followed by total answers provided. Results displayed in percentages. (PDF) [file pone.0300824.s003.pdf]

Table S2: Students' accommodations in semester 1, academic year 20/21

Where to you live this semester?	**Study 1 (Nov 20)**		
	**L4**	**L5**	**L6**
On campus	**34.6**	10.7	**25.0**
Private off-campus	19.2	**25.0**	**31.3**
at home (parents)	26.9	42.9	**25.0**
at home (own/with partner/with children)	19.2	17.9	18.8
prefer not to say	0.0	0.0	0.0

Explanations: Responses from the *Learn!Bio* Study 2 in April 2021, one month after the third national lockdown had come to an end. Multiple answers possible. Numbers in brackets after the level L4-L6 indicate the total participants followed by total answers provided. Results displayed in percentages.
