# Supplementary material for: Learn!Bio—A time-limited cross-sectional study on biosciences students’ pathway to resilience during and post the Covid-19 pandemic at a UK university from 2020–2023 and insights into future teaching approaches
Source: PLoS One. 2025 Sep 25;20(9):e0300824. doi: 10.1371/journal.pone.0300824 (PMC12463289; doi:10.1371/journal.pone.0300824)
Supplement: S2 Table — Responses from the Learn!Bio Study 2 in April 2021, one month after the third national lockdown had come to an end. Multiple answers possible. Numbers in brackets indicate the total participants followed by total answers provided. Results displayed in percentages. (PDF) [file pone.0300824.s004.pdf]

Table S4: New skills and talents developed during the Covid-19 pandemic.

Looking back at the past academic year: Do you believe you have developed or nurtured a wealth of skills or talents as a consequence of long times spent in lockdown? Please indicate if any of the below apply to you.	**Study 2 (Apr 21)**		
	**L4 (16/96)**	**L5 (11/34)**	**L6 (7/37)**
I am now (much more) computer and IT literate	50.0	27.3	71.4
I can fix many of the standard IT/internet/Microsoft Office problems myself	62.5	36.4	71.4
I am able to use frequently used conference software tools, including Blackboard Collaborate, MS Teams, and Zoom	87.5	72.7	57.1
I am more relaxed about smaller problems and hurdles - It is the whole picture which counts	**50.0**	**9.1**	**28.6**
I have learnt to skim read scientific books and papers– quickly skimming lots of literature in shortest time and still understanding its content.	68.8	36.4	85.7
I am much better with MS Office, including PowerPoint, Excel and Word.	75.0	54.5	57.1
I know how to seek remote support (e.g. from the department admin team, my personal tutor, or the Catalyst team)	68.8	45.5	85.7
I have learned to work self-paced towards a deadline and to plan ahead in a timely fashion	**56.3**	**27.3**	**71.4**
I can work remotely/virtually in teams, conducting group activities and group assessments.	**75.0**	**0.0**	**0.0**

Explanations: Responses from the *Learn!Bio* Study 2 in April 2021, one month after the third national lockdown had come to an end. Multiple answers possible. Numbers in brackets indicate the total participants followed by total answers provided. Results displayed in percentages.
